# Precision Breeding for a Global Staple Food: A Systematic Review with a Strategic Framework for CRISPR-Cas Applications in Rice (*Oryza sativa* L.)

**DOI:** 10.3390/genes17020165

**Published:** 2026-01-30

**Authors:** Nlhavat Gabriel Machel Gica, Wilard Tuto Gica, Honggui La, Yi Mi, Yi Zhou

**Affiliations:** 1 College of Life Sciences, Nanjing Agricultural University, Nanjing 210095, China; 2 Research Centre for Livestock Environmental Control and Smart Production, College of Animal Science and Technology, Nanjing Agricultural University, Nanjing 210095, China; 3 Zootechnical Engineering, Division of Agriculture, Instituto Superior Politécnico de Manica, Vandúzi District, Chimoio P.O. Box 113, Mozambique

**Keywords:** rice, CRISPR system, gene, resilience, trait prioritization framework, HDR efficiency, food security

## Abstract

**Background:** Rice is one of the world’s main staple crops, and improving its productivity and resilience is important to achieving food security under varying climatic conditions. **Objectives:** This systematic review synthesizes the existing evidence on the application, technical limitations, and potential of the development of genome editing technologies (CRISPR-Cas) in rice (*Oryza sativa* L.), as well as presents a novel approach called the CRISPR Trait Prioritization and Readiness Framework (CTPRF). **Methods:** Peer-reviewed articles that reported applications of genome editing based on the CRISPR-Cas system in the genome of rice for trait improvement or functional genomics were identified through searches fromPubMed, Scopus, Web of Science, and Google Scholar with studies published between 2012 and 2025. Studies were screened on predefined inclusion criteria related to experimental validation, reporting of editing efficiency, and clear phenotypic results. Data on CRISPR systems, target genes, methods of delivery, traits modified, and phenotypic results were extracted and synthesized by comparative analysis. **Results:** A wide variety of different CRISPR systems have been used in rice, and our results indicate that NHEJ-mediated knockouts are effective in average genotypes with editing efficiencies in the range of 70–90%, but HDR and prime editing are still under 10%. The CTPRF is being introduced as a strategic decision support tool to evaluate traits from four dimensions: technical feasibility, phenotypic predictability, impact potential, and regulatory pathway. We use this framework for case studies in pioneering countries (USA, Japan, China) and show how it can be useful for guiding research investment and policy. **Conclusions:** CRISPR-Cas technologies have transformed rice breeding, but their introduction requires overcoming genotype-dependent barriers to transformation and negotiating patchwork regulatory environments. The CTPRF offers a roadmap for the acceleration of the development of climate-resilient and nutritious rice varieties for the action plan.

## 1. Introduction

Rice (*Oryza sativa* L.) is one of the main staple food crops that provides over 50% of the world population’s caloric supply [1,2]. However, its productivity is under threat due to climate change-induced abiotic and biotic stresses, along with the reduction in arable land [1]. With the world population expected to rise to 9.1 billion by 2050 [3], there is a dire need for a major boost in productivity. Classical breeding approaches are usually time-consuming and constrained by genetic diversity, so they are not capable of dealing with immediate climate and demographic challenges [1,2,4].

The advent of CRISPR-Cas technology represents a paradigm shift in plant genomics and precision breeding [2]. This review goes beyond a listing of applications to offer a critical comparative analysis of editing strategies as well as their practical constraints. We systematically evaluate the relative merits of various types of CRISPR tools (Cas9, Cas12a, base editors, and prime editors) for particular kinds of trait modifications in rice. Furthermore, we propose a new method of prioritization of editing projects, namely a CRISPR Trait Prioritization and Readiness Framework (CTPRF), which is intended to serve as a guide to researchers, breeders, and decision makers in prioritizing editing projects based on technical feasibility, impact potential, and regulation.

This systematic review aims to (i) compare, in a critical way, the CRISPR-Cas editing modalities and their efficacy for different types of traits in rice; (ii) analyze the technical bottlenecks, paying particular attention to the recent progress to overcome HDR inefficiency and genotype-dependence; (iii) review the world regulatory landscape as well as present case studies on the early deployment; and (iv) introduce and apply the CTPRF to provide a strategic roadmap for the responsible and efficient development of CRISPR-edited rice varieties.

## 2. Materials and Methods

### 2.1. Literature Search Strategy and Study Selection

A systematic literature search was performed in PubMed, Scopus, Web of Science, and Google Scholar to identify the relevant studies related to genome editing in rice published from 2012 to 2025. The search was conducted with structured Boolean combinations of keywords which were related to the target species, genome-editing technologies, and important research results: (“*Oryza sativa*” or rice) and (CRISPR or Cas9 or Cas12a or “base editing” or “prime editing”) and (yield or stress or resistance or biofortification or “functional genomics”, were considered. Retrieved records were screened by title and abstract to exclude duplicates and non-relevant studies, then by full-text to ensure the research studies were relevant to agronomic traits, stress tolerance, nutritional improvement, or functional genomics, and only original, English-language research articles were included in the final analysis.

### 2.2. Eligibility Criteria and Screening Rationale

**Figure 1:**
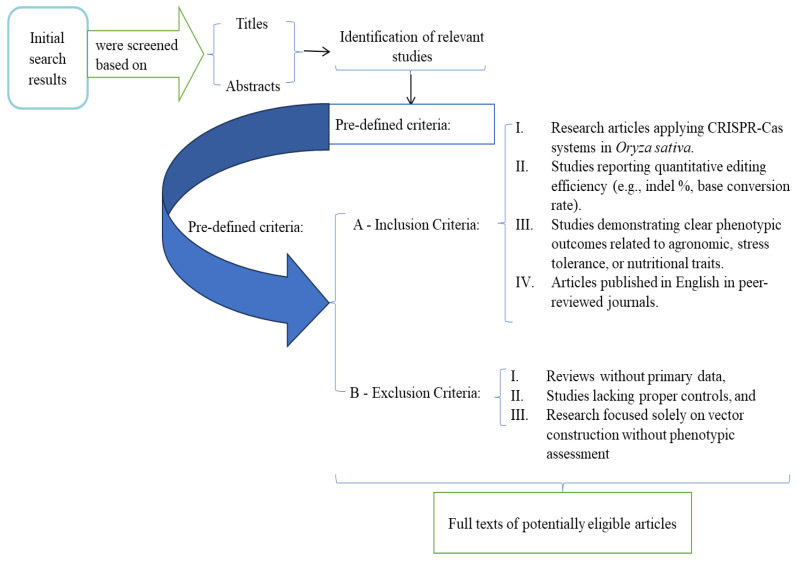
Study Selection and Eligibility Criteria.

The eligibility criteria used are illustrated in Figure 1. The inclusion criteria were designed to capture those studies with experimental validation with translational relevance. However, the exclusion criteria focused on the approach that ensured that the synthesis is built on robust, reproducible evidence, addressing the concern regarding the clarity of the conceptual rationale behind selection.

**Figure 2:**
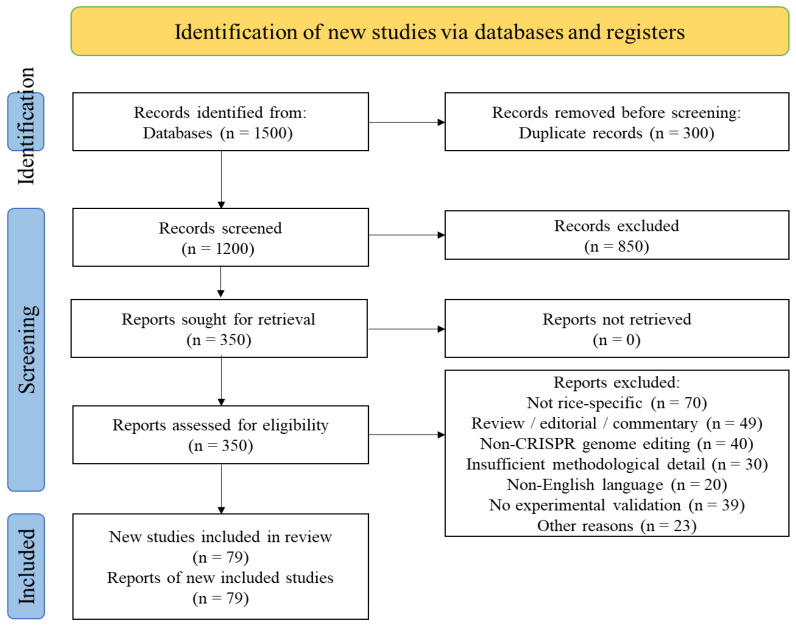
Study Selection Flowchart Following Prisma Guidelines.

Two authors independently screened titles, abstracts, and full texts. Discrepancies were resolved through consensus discussion. A formal risk-of-bias assessment was not conducted due to the methodological heterogeneity and experimental nature of the included studies. The study selection process followed PRISMA 2020 guidelines (Supplementary Materials Section), as well as illustrated in Figure 2.

### 2.3. Data Extraction and Synthesis

Data were gathered with the use of a standardized extraction template and organized in a systematic manner to enable meaningful comparison between studies. Information collected from each publication included the genome-editing system and strategy used, such as the specific nuclease used and editing strategy used, for instance, NHEJ, HDR, base editing, or prime editing. Technical performance indicators such as editing efficiency, transformation method, and rice genotype used were also noted. In addition, studies were categorized based on trait category, including yield and grain quality, biotic and abiotic stress tolerance, nutritional biofortification, and functional genomics, and the associated phenotypic outcome was recorded as a quantitative change in traits, including yield improvement, reduction in disease severity, or change in nutrient concentration. Data synthesis was performed using comparative analysis by classifying studies by editing strategy and category of trait to look for patterns of efficiency, success rates, and recurring technical limitations. This structured approach enabled the review to progress beyond the description of the different genome-editing strategies and move towards a critical appraisal of the strengths and weaknesses of the various strategies and their practical implications for rice.

#### Protocol and Registration

This systematic review was conducted in accordance with the PRISMA 2020 guidelines. The review protocol was not registered in PROSPERO.

## 3. Results and Critical Analysis

### 3.1. Study Selection and Characteristics

Out of one thousand and five hundred (1500) identified records, seventy-nine (79) studies met the inclusion criteria (Figure 2).

### 3.2. Comparative Analysis of Editing Modalities: Efficacy and Constraints

A critical synthesis of the extracted data reveals distinct efficiency profiles and optimal use cases for each CRISPR modality in rice (Table 1).

#### 3.2.1. NHEJ-Mediated Knockouts

NHEJ-mediated gene knockout is the most established and technically robust genome-editing approach in rice, which have been reported in japonica cultivars such as Nipponbare using Agrobacterium-mediated transformation [5,6,7,8,9], on the other hand, a specific human cell based study, reached a maximum efficiency of 75% [10] and also a mice cell based study reported an efficiency of 90% [11], which shows its high efficiency even in other species. Most of the editing events that were reported for modifying a plant’s genome were performed through error-prone NHEJ [12]; NHEJ is efficient but is inherently error-prone and generates random insertions or deletions that render the results not predictable [13,14].

#### 3.2.2. Base Editing (CBE/ABE)

In Japonica rice varieties such as Nipponbare, the base editing has achieved phenomenally high transformation efficiencies in the range of 88.9–100% [9,15]. This technology is especially useful in producing alleles of herbicide resistance [16,17] or mutations of specific amino acids to fine-tune protein performance [18]. One of its major advantages is that it does not induce double-strand breaks, and so decreases unintentional insertions or deletions compared with traditional CRISPR/Cas9 knockouts [19,20]. Base editing has been applied to precisely modify important genes in rice, and has allowed the creation of valuable rice traits with agronomic potential, such as herbicide tolerance and improved nitrogen use efficiency [21]. However, despite these strengths, the method is limited by a narrow editing window and a protospacer-adjacent motif (PAM) in the correct orientation, which limits the number of loci that can be effectively targeted [20,22].

#### 3.2.3. Homology-Directed Repair (HDR)

As always pointed out, HDR efficiency is super low, 10%, and is the major bottleneck for accurate gene replacement/insertion [23,24,25,26]. This means that HDR has low efficiency as compared with the NHEJ pathway [27]. Recent attempts to solve this problem involve the use of geminivirus replicons as donor templates [26] and the manipulation of the endogenous repair mechanisms, but these are experimental and genotype specific. Nonetheless, the HDR-based genome editing remains highly challenging and inefficient in plants, and the primary reason for this is the low incorporation efficiency of the donor DNA template into the plant cells [28].

#### 3.2.4. Prime Editing

Prime editing in rice has also shown initial promise with high editing efficiencies in some of the studies, varying between 77.08% to 88.5% in optimized conditions at the specific target loci [29,30]. However, in spite of these encouraging results, in most reports, in rice, very low overall editing efficiencies and challenges in recovering stable transgenic lines with heritable edits have been reported, indicating current limitations of the technology [31,32]. These constraints may be overcome by engineering alternative/improved reverse transcriptase enzymes in order to improve editing performance [33] and optimizing the selection of transgenes and expression systems, which may improve the recovery of stable edited plants [34]. With continued improvement in the methodology, prime editing could potentially be an effective tool for precision molecular breeding to be employed in crop improvement programs.

**Table 1:** Critical comparison of CRISPR-Cas editing modalities in rice.

Editing Modality	Primary Mechanism	Reported Efficiency	Representative Use Cases	Major Constraints	Sources
NHEJ-Mediated Knockout	Error-prone repair of DSBs generating indels that disrupt gene function	High (commonly >70% in rice; up to 75–90% in mammalian systems)	Loss-of-function traits, including knockout of negative regulators and susceptibility genes	Inherently error-prone; unpredictable indel size; genotype-dependent transformation efficiency	[5,6,7,8,10,11,12,13,14]
Base Editing (CBE/ABE)	Direct single-nucleotide conversion without DSB formation	High transformation efficiency (up to 100% in Nipponbare); variable editing frequency	Herbicide resistance alleles and precise amino acid substitutions	Restricted editing window; strict PAM requirement; limited to specific base transitions	[9,16,17,20,22]
HDR-mediated Editing	Donor-template-guided homology-directed repair	Low (generally <10%)	Precise gene replacement or targeted gene insertion	Extremely low donor DNA incorporation; strong competition with NHEJ; genotype dependence; technically demanding	[23,24,25,26,28,34]
Prime Editing	Reverse transcriptase-mediated DNA synthesis guided by pegRNA without DSBs	Highly variable: <1% in many studies; up to 77.08–88.5% at specific loci under optimized conditions	Precise base substitutions, small insertions, and deletions for molecular breeding	Low reproducibility; difficulty recovering stable transgenic lines; large construct size; extensive optimization required	[29,30,31,32,33,34]

### 3.3. Analysis of Key Trait Categories and Editing Success

#### 3.3.1. Yield and Grain Quality

Editing of traits that are associated with yield has been especially successful in rice because the size and weight of the grain are determined by well-characterized negative regulatory genes. In particular, multiplex knockouts of, for example, *GS3*, *GW2*, and *GW5*, which act to limit grain elongation and mass normally, thus allow breeders to simultaneously eliminate multiple genetic limitations in one editing step [35,36]. By simultaneously targeting multiple loci, this approach cancels out the effects of genetic redundancy and has additive phenotypic effects that are converted into obvious yield improvements. Consequently, edited rice lines have exhibited 28–30% of grain weight and yield per panicle increase compared against wild-type controls, reflecting the robustness of this approach [37,38,39].

#### 3.3.2. Biotic Stress Resistance

Improvement of biotic stress resistance of rice has been achieved with a certain success by knockout of promoter elements in the *OsSWEET* family of genes that provide durable, broad-spectrum resistance to bacterial blight, a major yield-limiting disease [40,41,42]. By interfering with the particular susceptibility elements exploited by pathogens, this approach provides predictable and stable resistance without interfering with other plant functions. Within the CRISPR Trait Prioritization and Feasibility framework (CTPRF) for rice breeding, this falls within the first quadrant, the Rapid Deployment trait categorization, as it provides both high technical feasibility and consistent beneficial outcomes and can therefore be implemented rapidly by breeders in elite varieties [43,44,45,46].

#### 3.3.3. Abiotic Stress Tolerance

Enhancing abiotic stress tolerance in rice has experienced some great success by genome editing of negative regulators, such as *OsRR22*, which provides salinity tolerance [8,46]. However, traits such as drought tolerance are governed by complicated, polygenic networks and hence are more difficult to engineer. Multiplex editing strategies, which target multiple genes at the same time, represent a very promising route to tackle these complicated traits; however, their efficiency decreases with the number of guide RNAs. Therefore, it represents an existing technical challenge when it comes to engineering polygenic stress responses [47,48,49].

#### 3.3.4. Nutritional Biofortification

**Figure 3:**
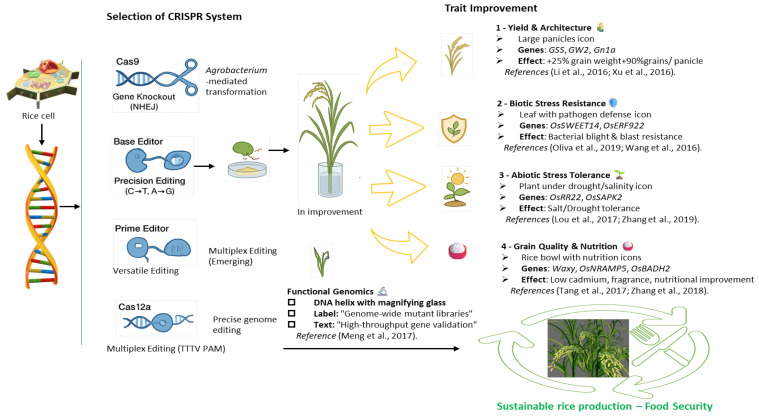
Application of CRISPR-Cas in Rice (*Oryza sativa*) Molecular Biology [8,38,40,51,56,57,58,59,60].

Efforts to improve the nutritional quality of rice have seen some success, such as knocking out *OsNRAMP5* to reduce cadmium accumulation in grains [50,51,52]. However, high levels of allelic variation in rice and maize that influence nutrient accumulation and metabolism are problematic for gene discovery and genetic manipulation. Harnessing this natural diversity necessitates advanced molecular tools and high-throughput phenotyping platforms as well as extensive functional genomics studies, and cereal genome complexity is a major technical bottleneck for nutritional enhancement [53]. Enhancing key minerals such as iron and zinc will often require an increase in transporters or chelators, and may require precise HDR-based editing or gene activation strategies, putting these traits in a more complex, technically challenging category [54,55]. While the potential benefits of application of the genome-editing tools (including CRISPR-Cas9) to rice grains in Fe and Zn biofortification have important properties, they remain largely unexplored, providing an important avenue for future research [55] (Figure 3).

### 3.4. The CRISPR Trait Prioritization and Readiness Framework (CTPRF)

**Figure 4:**
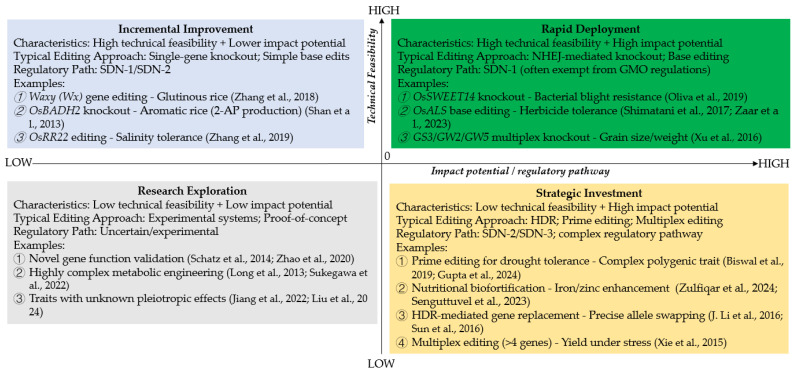
The CRISPR Trait Prioritization and Readiness Framework (CTPRF) for Rice Breeding [1,8,16,17,25,26,38,40,45,48,61,62,63,64,65,66,67,68,69].

Figure 4 shows the Trait Prioritization and Readiness Framework for Rice Breeding using the CRISPR Trait Prioritization and Readiness Framework (CTPRF). Quadrant I (Rapid Deployment) contains high-priority traits with well-established editing methods and clear agronomic or nutritional benefits, e.g., knockout of *OsSWEET14* for bacterial blight resistance. Quadrant II (Incremental Improvement) contains those traits that are technically feasible but have a moderate impact. Quadrant III (Research Exploration) consists of the traits that require a great deal of foundational research before they can be put to use. Quadrant IV (Strategic Investment) comprises high-impact characteristics that are held back today by technical constraints, such as the use of prime editing in the improvement of complex drought tolerance. Dashed arrows show possible migration of traits between quadrants with the advance of both the technology of CRISPR and understanding of biology, combined with the growing complexity of regulation.

### 3.5. Application to Case Studies

#### 3.5.1. Japan: Herbicide-Tolerant Rice

In Japan, herbicide-resistant rice has been developed by base editing to induce precise point mutations in the *OsALS* gene, which encodes acetolactate synthase, an important target of several herbicides [16]. These types of edits change the area where the herbicide binds to the enzyme without disrupting normal enzyme activity so that plants can take in the herbicide and still grow and produce at normal levels. Because the double-strand breaks and insertion of foreign DNA are not required in base editing, the resultant plants exhibit high levels of genetic stability and predictable phenotypic results [19]. In addition to the technical benefits of this approach, the success of this approach is tightly connected to the Japanese regulatory environment. The fact that SDN-1 gene-edited crops are not classified as GMOs has meant that regulatory complexity is lower, timelines are shorter, and there is more interest in more broadly adopting base-editing strategies. Together, these factors make herbicide-tolerant rice in Japan an obvious example of the synergistic effects of precise genome editing and supportive regulation to boost crop improvement [70].

#### 3.5.2. United States: High-Yield Rice

In the United States, the improvement of rice yield has depended on the use of multiplex genome editing to target multiple genes at once, such as *GS3*, *GW2*, and *GW5*, which are genes that, naturally, limit the size and weight of the grain [38]. By editing several loci simultaneously, breeders are able to create additive effects with significant yield improvement, although the approach is technically more difficult than when working with single genes. This strategy falls into the category of the Strategic Investment trait because even though the technical demands are higher, the potential agronomic reward is great. The USDA’s product-focused and flexible regulatory framework further enables this approach and will enable edited lines to be subjected to field trials, enabling the testing, optimization, and ultimate deployment of high-yield varieties at a faster pace [71].

#### 3.5.3. China: Disease-Resistant Rice

In China, scientists have enhanced rice resilience by knocking out the *OsSWEET14* gene, a gene normally responsible for making plants prone to bacterial blight, one of the most destructive diseases that can wipe out yields [40]. This specific approach gives good, strong, and reliable resistance without having a negative effect on growth or productivity. Because the method is simple and the results are very predictable, it is said to be a Rapid Deployment trait, which means that it can be rapidly used in breeding programs. China’s regulatory environment has also been becoming more favorable for gene-edited crops. Crops without foreign DNA have more simplified approval processes, as researchers can transition from the lab to the field and potentially to commercialization in a shorter timeframe [72]. When combined, the power of advanced genome-editing technology and supportive regulations is making disease-resistant rice an obvious example of how scientific innovation can translate into tangible and practical benefits to the farmer in a relatively short time.

### 3.6. Technical Challenges and Recent Mitigation Strategies

#### 3.6.1. Overcoming HDR Inefficiency

Homology-directed repair (HDR) is one of the least efficient plant genome-editing pathways [27], and strategies to overcome the limitation of HDR have begun to be developed. Recent approaches involve the use of viral replicon systems [73], in order to increase the intracellular copy number of the donor templates and thereby increase the chances of HDR-mediated repair events [23,26]. Additional efforts have been directed at co-expression of key genes in the HDR pathway (including factors such as *RAD54*), to favor the repair of DNA to precise recombination as opposed to error-prone NHEJ [24,74]. Furthermore, the development of synchronization strategies of the cell cycle to enrich the S and G2 cells has been attempted to enrich cells that have a naturally increased activity of HDR [75,76].

#### 3.6.2. Addressing Genotype-Dependent Transformation

Genotype-dependent transformation is still a major constrain especially in the case of elite indica rice varieties that are highly recalcitrant for regeneration [77,78]. Current strategies to meet this challenge are improving Agrobacterium strains and co-cultivation conditions, cultivar-specific [79], the use of morphogenic regulators (Baby Boom and Wuschel) to improve regeneration capacity in recalcitrant genotypes [80,81], and the use of different modes of delivery (nanoparticle-mediated or pollen-tube pathway transformation) [82,83]. However, these alternative approaches are still mainly experimental and are still less reliable for the successful attainment of stable and heritable transformations than the conventional Agrobacterium-mediated systems.

#### 3.6.3. Manipulating Polygenic Traits

Improvement of traits that are controlled by multiple quantitative trait loci (QTLs) requires the use of multiplex genome editing, because in many cases, single-gene modification is not sufficient to generate meaningful phenotypic improvements. To improve the efficiency of multiplexing, a few novel of approaches have been developed, such as tRNA–gRNA array systems, where multiple guide RNAs can be simultaneously expressed and accurately processed from a single transcript [48], and also Csy4-based polycistronic approaches, where RNA endonuclease-mediated cleavage is employed to generate functional gRNAs, with a high degree of accuracy [84]. A key observation from our analysis is that editing efficiency decreases by large margins for >4 targets, which may indicate an upper bound for existing technology.

### 3.7. Regulatory Landscapes

Regulatory frameworks for gene-edited crops vary considerably between countries as a result of differences in policy priorities and ways of perceiving the risks. Argentina adopted a specific regulation for gene-edited crops in 2018, treating products made through SDN-1 as non-GM and products through SDN-3 as GM, but without any regulatory framework for SDN-2 products [85]. In Australia, organisms developed through SDN-1 techniques are not subject to the existing GMO regulations, while in Canada, organisms developed via both SDN-1 and SDN-2 techniques are exempt from the regulations, under its product-based regulatory system [86]. Similarly, the application of SDN-3 is regulated in Brazil and Argentina, but generally not for SDN-1 and SDN-2 edits that do not lead to recombinant DNA in the final product [87,88]. In China, the Ministry of Agriculture and Rural Affairs issued some official safety evaluation guidelines for gene-edited crops at the beginning of 2022. Under this framework, the gene-edited crops without foreign DNA are still formally under the GMO regulatory system but are subject to simplified and accelerated food and environmental safety assessments compared with conventional GM crops, suggesting a rather supportive but cautious regulatory attitude [72].

## 4. Conclusions and Future Perspectives

CRISPR-Cas technology has obviously changed the landscape of rice research with new possibilities to improve this very important crop with a level of precision that was not possible in the past. Rather than merely summarizing existing studies, the present review is designed to draw together the current state of the art, provide a critical comparison of approaches available, and identify a practical framework (CTPRF) to guide future applications.

Looking into the future, progress will be dependent on four interrelated priorities. First, there is a need to further improve genome-editing tools, especially by improving the efficiency of HDR and prime editing, and developing transformation methods that are functional in elite varieties of rice. Second, trait selection should be more strategic, utilizing frameworks such as the CTPRF, in order to balance traits that can provide short-term gains, such as disease resistance, and more complex and long-term goals, such as tolerance to multiple environmental stresses. Third, greater interaction with policymakers is necessary to help promote science-based and proportionate regulations that unambiguously distinguish precision-edited crops from traditional transgenic organisms. Finally, it will be the responsible translation that leads to successful real-world impact, where societal acceptance, ethics, and stakeholder perspectives are taken into consideration right from the start of product development.

By taking this integrated and thoughtful approach, the scientific community can ensure that the use of these technologies (CRISPR-Cas) will be used responsibly and effectively, which will help build a more sustainable, resilient, and food-secure future.

## Data Availability

No new data were created or analyzed in this study. Data sharing is not applicable to this article.

## Supplementary Materials

The following supporting information can be downloaded at: https://www.mdpi.com/article/10.3390/genes17020165/s1: PRISMA 2020 checklist. Reference [89] is cited in Supplementary Materials.
